# Reimagining Dementia Care with Soft Robotics: Unveiling Patient Needs to Inspire Innovative Design Frameworks

**DOI:** 10.1002/alz70858_106705

**Published:** 2025-12-25

**Authors:** Anne Roudaut, Bijetri Biswas

**Affiliations:** ^1^ University of Bristol, Bristol, United Kingdom

## Abstract

**Background:**

Soft robotics holds promise in healthcare, enhancing rehabilitation and daily support for physical and mental well‐being. Despite this potential, their role in caring for older adults with neurodegenerative disorders (NDD) remains underexplored, even though the World Health Organization identifies NDD as a major global health challenge. User acceptance is critical but shaped by complex factors that designers often struggle to access and apply effectively.

**Method:**

This study explores perceptions of soft robotics for NDD care, focusing on usability, safety, emotional comfort, and design preferences. Data were gathered from focus groups, interviews, and co‐design sessions with patients, caregivers, and clinicians. Discussions were facilitated using a virtual think‐space and structured co‐design workshops.

**Result:**

Participants expressed a strong preference for large‐scale soft robotics, viewing them as engaging, comforting, and immersive. They emphasised the need for multi‐functional designs integrating physical assistance, sensory stimulation, and emotional engagement. Larger robots were seen as fostering a sense of security, encouraging social interactions, and strengthening relationships between patients and caregivers.

Concerns about safety, durability, and ease of maintenance were raised, highlighting the need for soft yet robust materials. Biomorphic designs inspired by nature were favoured for their emotional and aesthetic appeal. Clinicians also stressed the importance of modularity, allowing for customisable features to meet individual needs.

**Conclusion:**

Findings emphasise the importance of large‐scale, interactive, and adaptable soft robotics. By addressing user‐centred concerns, designers can enhance technology acceptance, foster empathy and ethical awareness, and create more engaging, supportive robotic solutions for both patients and caregivers.

